# Supplementary material for: Volatile Organic Compounds Emitted by the Biocontrol Agent Pythium oligandrum Contribute to Ginger Plant Growth and Disease Resistance
Source: Microbiol Spectr. 2023 Aug 3;11(4):e01510-23. doi: 10.1128/spectrum.01510-23 (PMC10433877; doi:10.1128/spectrum.01510-23)
Supplement: Supplemental file 3 — Table S3 and Fig. S1 to S10. Download spectrum.01510-23-s0001.pdf, PDF file, 1.8 MB [file spectrum.01510-23-s0001.pdf]

Table S1. (Separate Excel file) List of primers used for qPCR work. As well as the sequences, the Pfam annotations for ginger genes and gene description for *N. benthamiana*, primer efficiency value and the purpose are listed for each of the primer pairs.

Table S2. (Separate Excel file) **(A)** Transcriptomic dataset for ginger either exposed to the volatile organic compounds (VOCs) produced by *Pythium oligandrum* or non-exposed (control) at 21 d. Summary of gene models and functional annotations for ginger genes and the RNA-seq dataset. **(B)** Based on the Pfam annotations, the list of upregulated ginger genes related to growth and stress responses in plants either exposed to the volatile organic compounds (VOCs) produced by *Pythium oligandrum* or non-exposed (control) at 21 d.

Table S3. Screening for the identification of two growth-promoting VOCs, hexadecane and 3-octanone in *P. oligandrum* and *P. myriotylum*.

**VOCs** [a]	**Chemical Formula**	**RI (Exp.)**[b]	**RI (lit.)**[c]	***P. oligandrum* GAQ1**[d]	***P. oligandrum* CBS 530.74**[e]	***P. myriotylum* SWQ7**[f]
**3-octanone**	$C_8H_{16}O$	986	989	+	-	-
**hexadecane**	$C_{16}H_{34}$	1601	1600	+	+	-

a VOCs that promoted *N. benthamiana* growth were screened for the presence of these VOCs in other *Pythium* species.
b Retention index of experimental data.
c Retention index listed in the literature.
d *P. oligandrum* GAQ1 produced these two VOCs identified by (Sheikh et al., 2023).
e & f evaluated to produce two growth-promoting VOCs in the present study.

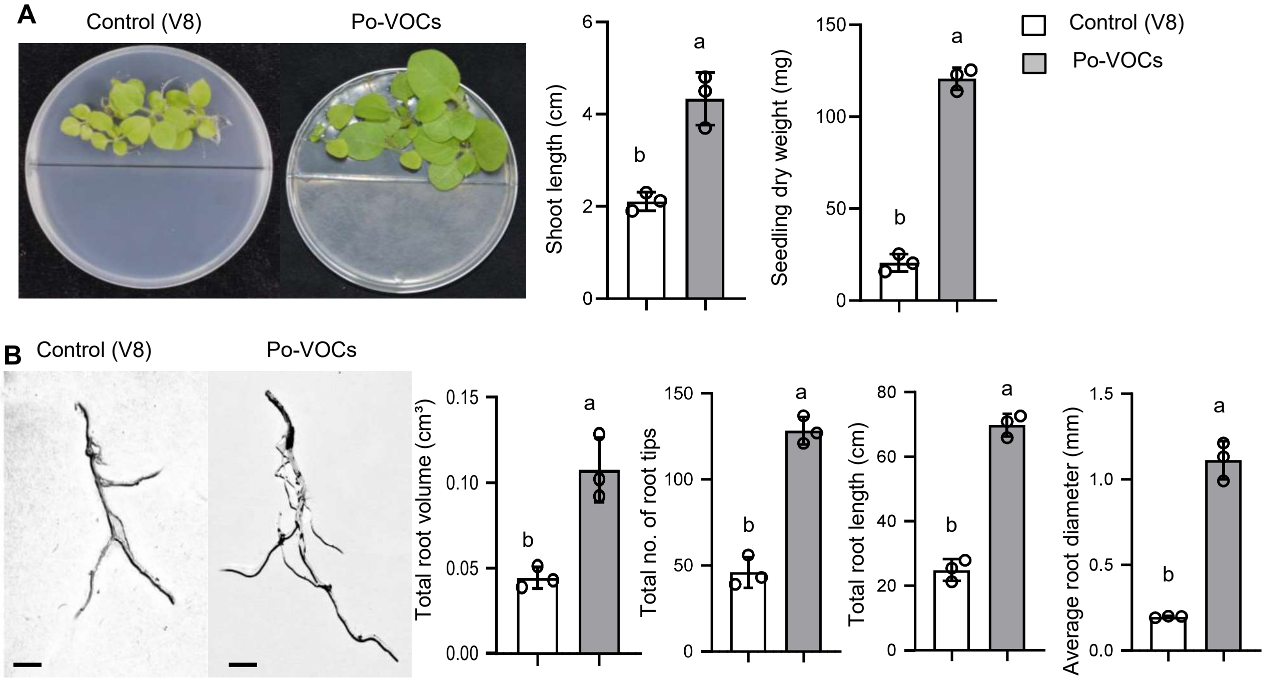

Figure S1. The results of the second repeat experiment also showed that *P. oligandrum* volatile organic compounds (Po-VOCs) enhanced the growth of *Nicotiana benthamiana* seedlings. **(A)** The plates were imaged 10 d after exposure to Po-VOCs or control (V8) and shoot length and seedling dry weights were measured. **(B)** Representative images of the roots of *N. benthamiana* seedlings exposed to Po-VOCs or control (V8) for 10 d (Scale bar represents 2 cm). Four root morphological parameters of total root volume, total number of root tips, total root length, and average root diameter were measured. Error bars indicate the standard error of the mean (n = 3). Different lower-case letters above the bars represent significant differences between treatments.

2nd repeat

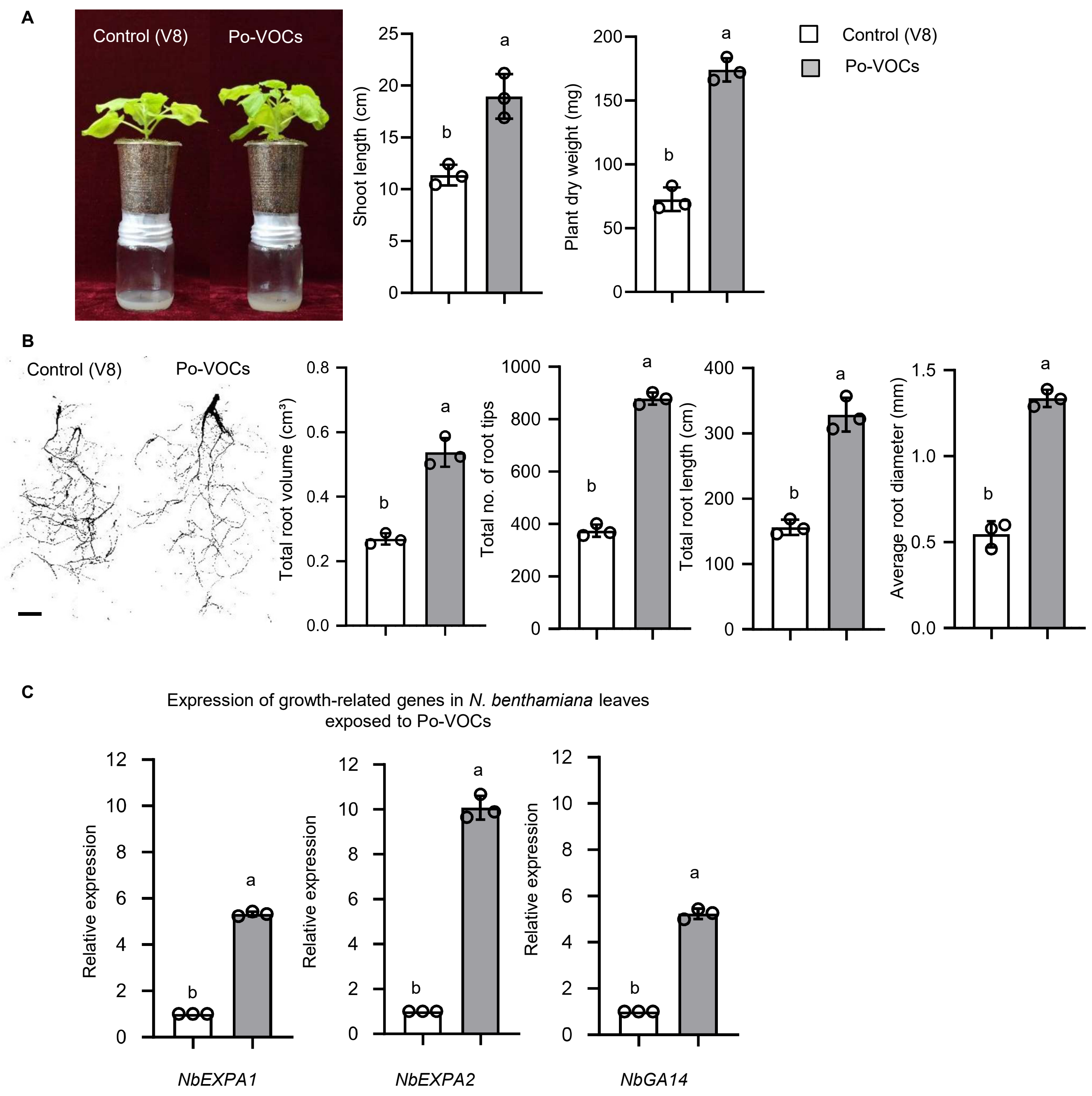

Figure S2. The second and third repeats of the experiment also showed that the volatile organic compounds produced by *P. oligandrum* enhanced the growth of *Nicotiana benthamiana in planta*. **(A and D)** The plants were imaged 21 d after exposure to Control (V8) or Po-VOCs to see the difference in growth by measuring the shoot length, and dry weight of the plants. **(B and E)** Representative images of the roots of *N. benthamiana* plants exposed to Po-VOCs or control (V8) for 21 d (Scale bar represents 2 cm). Root morphological parameters, total root volume, the total number of root tips, total root length, and average root diameter were measured. **(C and F)** Expression of the genes related to growth in leaves of *N. benthamiana* after 21 d of exposure of plants to the VOCs produced by *P. oligandrum*. qRT-PCR was performed using *NbEF-1a* as an internal reference. Error bars indicate the standard error (n = 3). Different lower-case letters above the bars represent significant differences between treatments. For D, E and F parts, see the next page.

3rd repeat

D

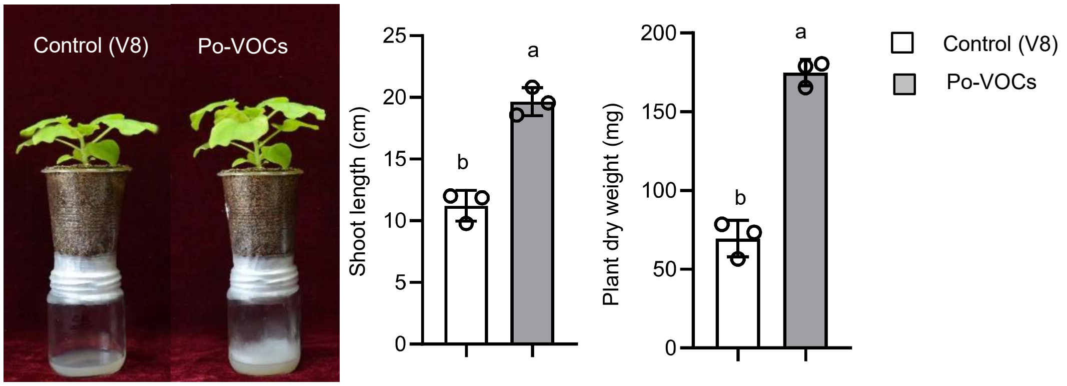

E

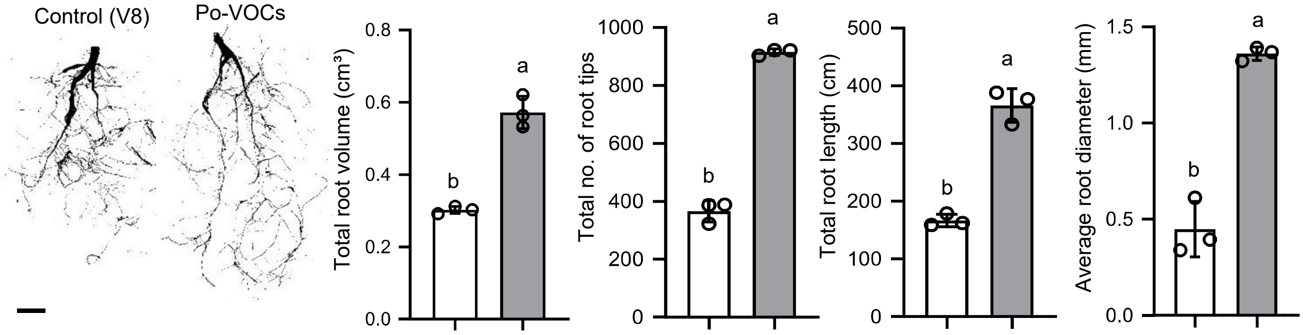

F

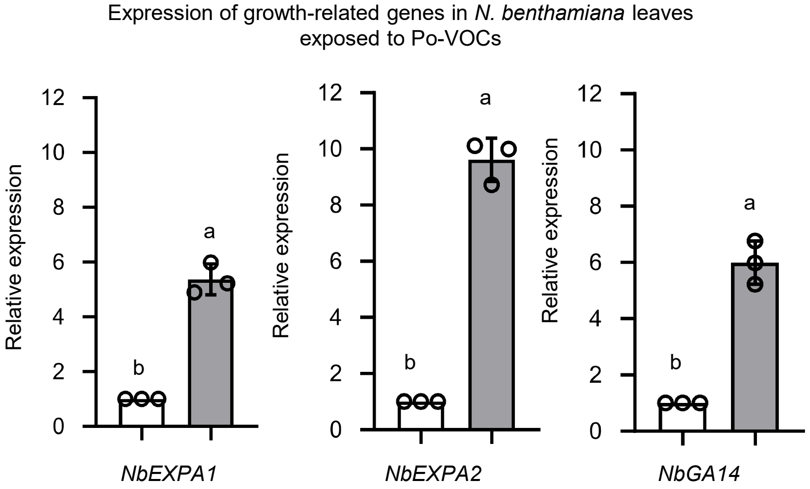

Figure S2. (continued from previous page) The second and third repeats of the experiment also showed that the volatile organic compounds produced by *P. oligandrum* enhanced the growth of *Nicotiana benthamiana in planta*. **(A and D)** The plants were imaged 21 d after exposure to Control (V8) or Po-VOCs to see the difference in growth by measuring the shoot length, and dry weight of the plants. **(B and E)** Representative images of the roots of *N. benthamiana* plants exposed to Po-VOCs or control (V8) for 21 d (Scale bar represents 2 cm). Root morphological parameters, total root volume, the total number of root tips, total root length, and average root diameter were measured. **(C and F)** Expression of the genes related to growth in leaves of *N. benthamiana* after 21 d of exposure of plants to the VOCs produced by *P. oligandrum*. qRT-PCR was performed using *NbEF-1a* as an internal reference. Error bars indicate the standard error (n = 3). Different lower-case letters above the bars represent significant differences between treatments. For A, B and C parts, see the previous page.

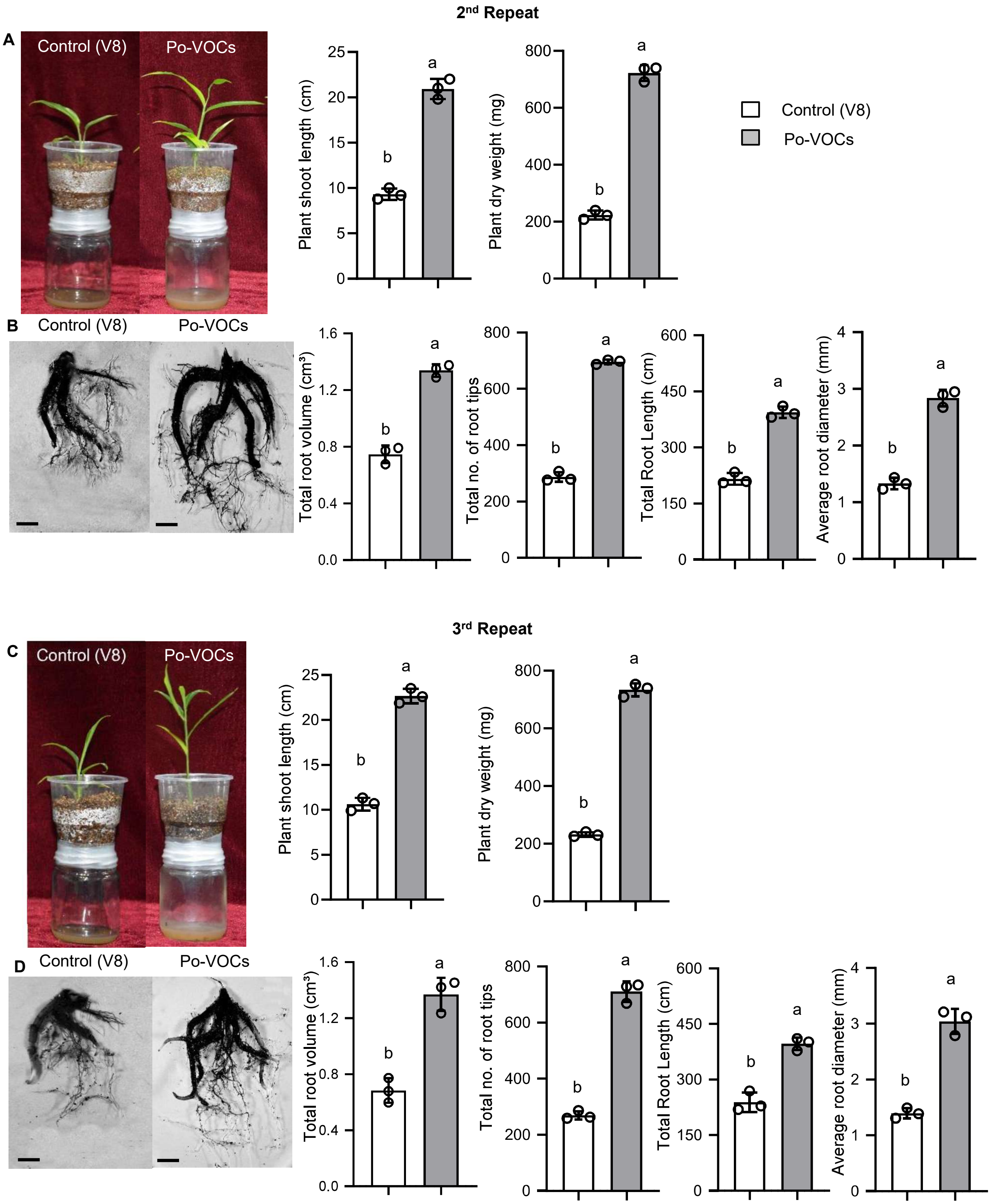

Figure S3. The second and third repeat of the experiment also showed that the volatile organic compounds produced by *P. oligandrum* enhanced the growth of ginger *(Zingiber officinale)* plants. **(A and C)** The plants were imaged 21 d after exposure to Po-VOCs or Control (V8) to see the difference in growth by measuring the shoot length, and dry weight of the plants. **(B and D)** Representative images of the roots of ginger plants exposed to Po-VOCs or Control (V8) for 21 d (Scale bar represents 2 cm). Root morphological parameters, total root volume, the total number of root tips, total root length, and average root diameter were measured. Error bars indicate the standard error (n = 3). Different lowercase letters above the bars represent significant differences between treatments.

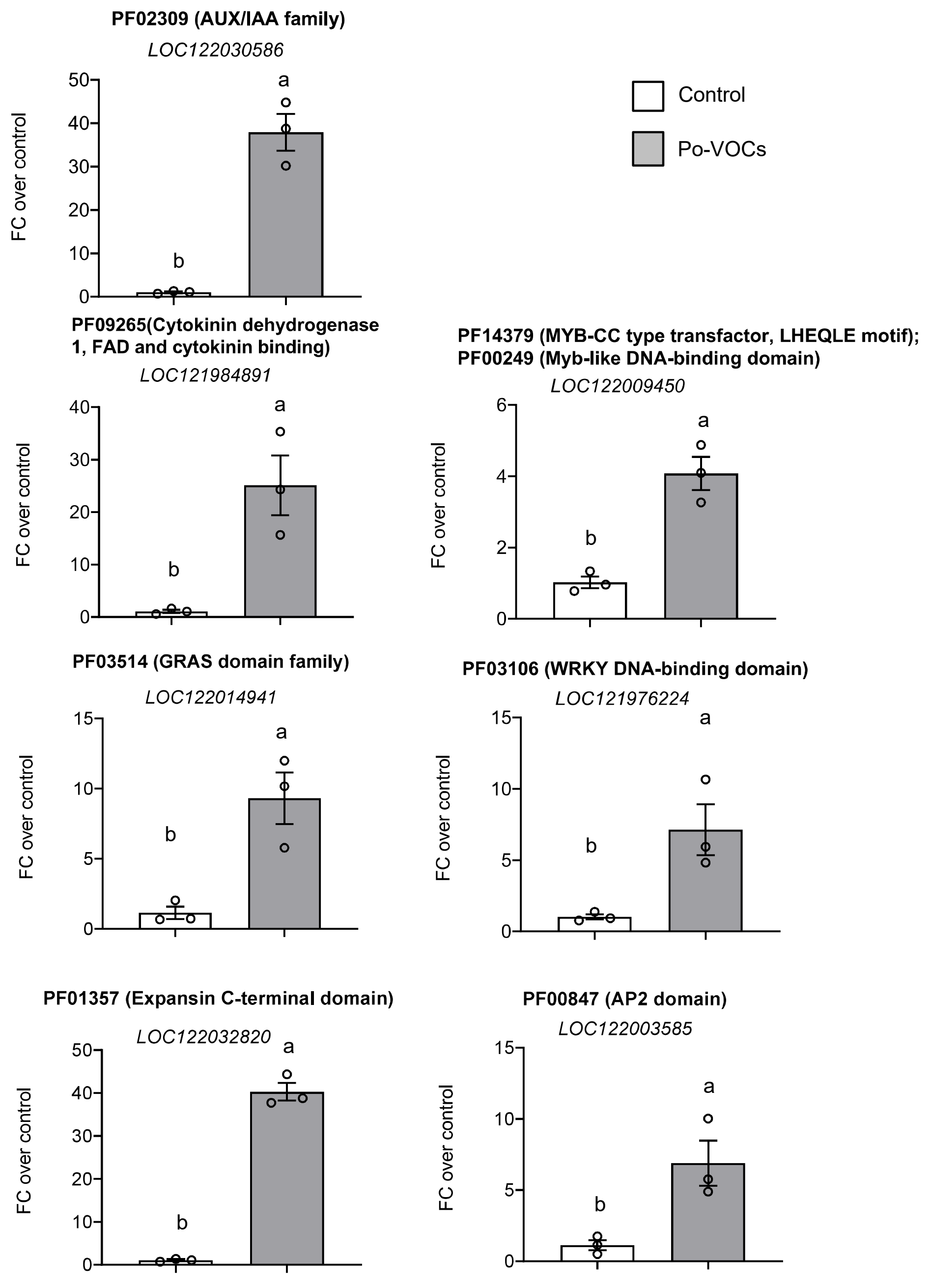

Figure S4. qPCR validation of RNA-seq dataset in ginger plants either exposed to Po-VOCs or control (V8). The genes related to growth and stress were selected based on the Pfam annotations. The error bars represent standard errors (n = 3). The expression levels of ginger were normalized using *ZoEF-1α* as a reference gene. Different lower-case letters above the bars represent significant differences between treatments using Student's t-test at the level of $P < 0.001$.

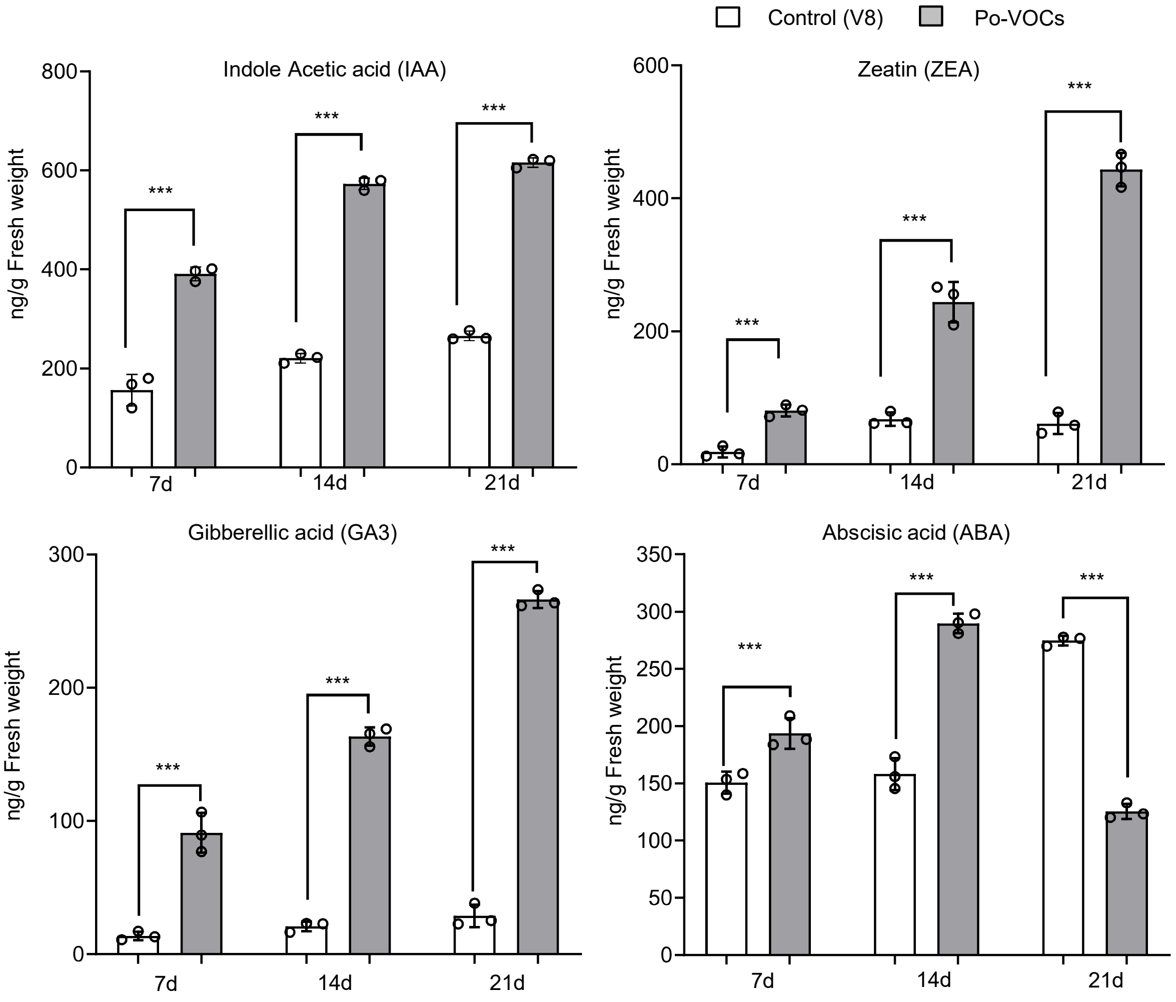

Figure S5. The results of the second repeat of the experiment also showed the trends similar to Figure 5 and showed the concentration of selected plant growth hormones in leaf blades of ginger plants exposed to *P. oligandrum* volatile organic compounds for 7 d, 14 d and 21 d. Error bars indicate the standard error (n = 3). *** represents the significant difference between treatment and control at the level of $P < 0.001$.

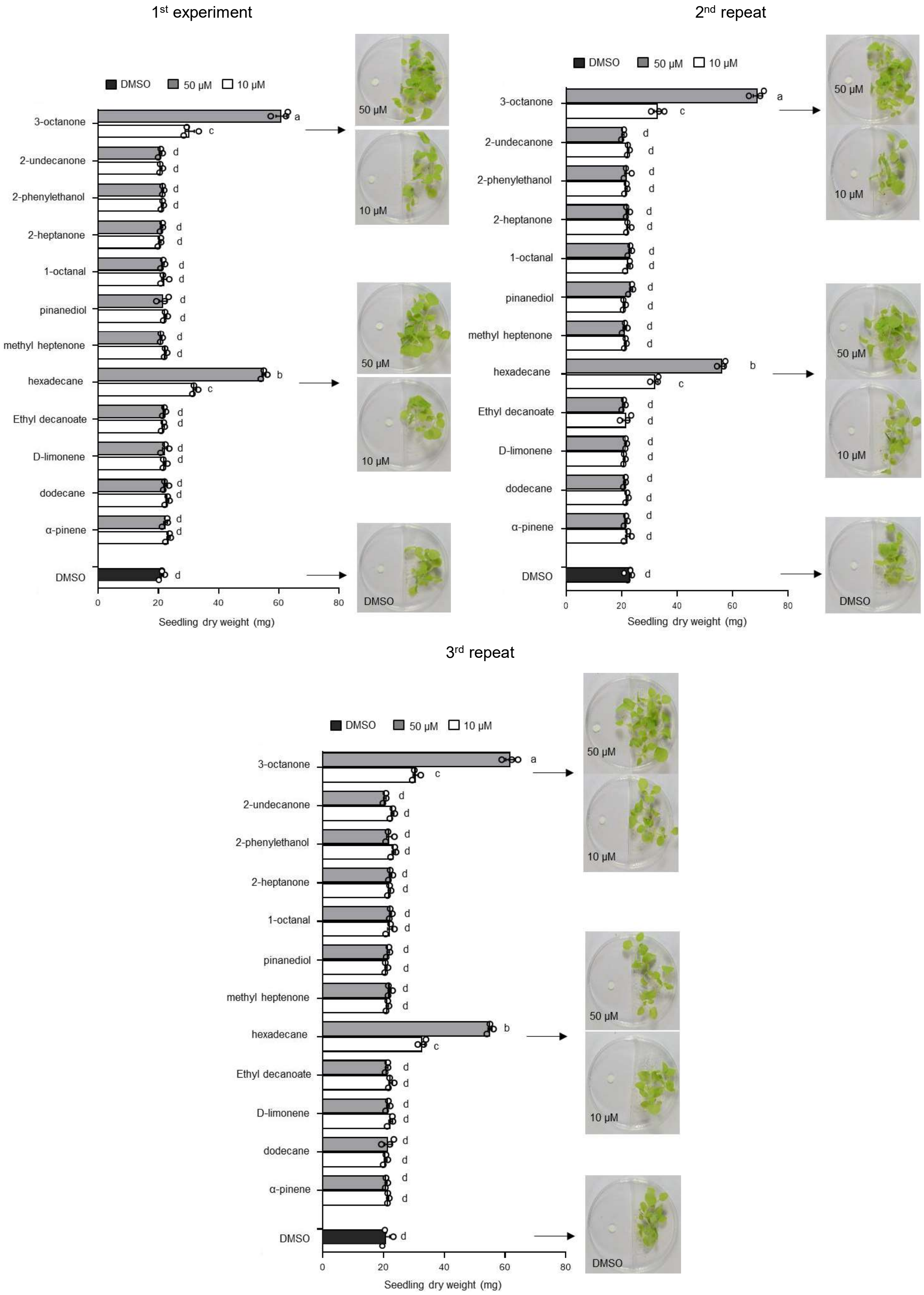

Figure S6. Assessment of the effects of individual VOCs produced by *P. oligandrum* (GAQ1) for their potential in growth promotion in *N. benthamiana* seedlings. Plant dry weights were recorded after 10 d of VOCs exposure at 10 µM and 50 µM concentrations and the Petri plates supplemented with DMSO were used as control. Error bars indicate the standard error of the mean (n = 3). The letters above the bars indicate a significant difference between conditions. Experiments were replicated at least three times producing similar results

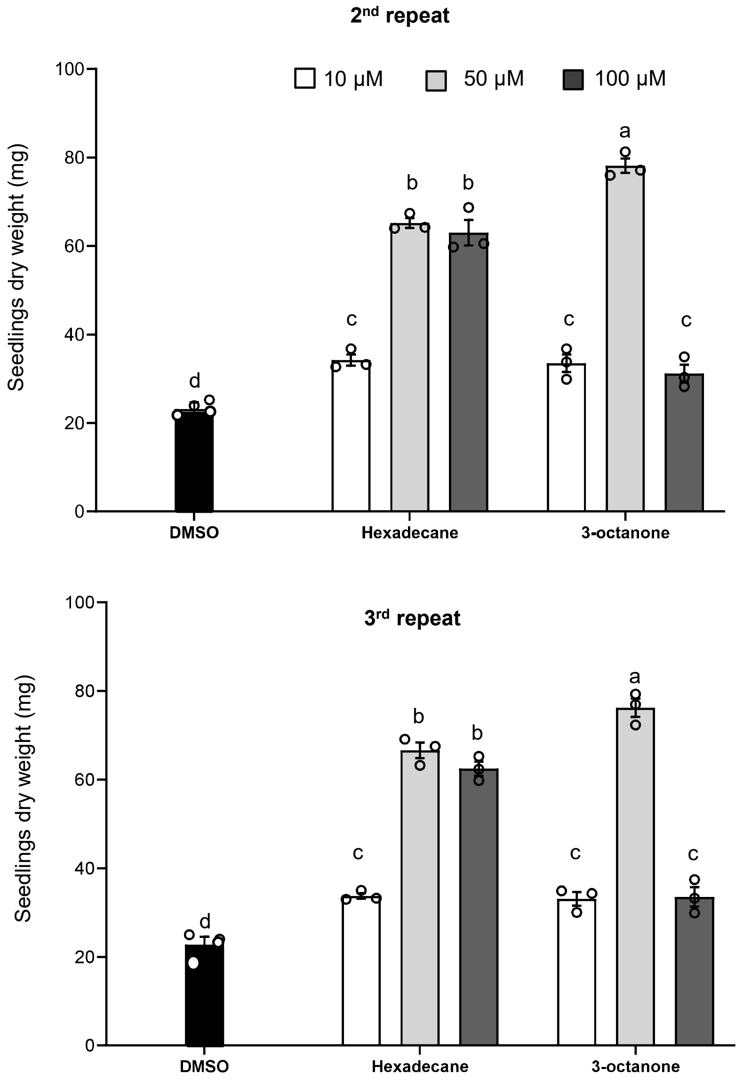

Figure S7. The results of the second and third repeat of the experiments showed the same trends as in Figure 6 showing the effect of two growth-promoting VOCs at a higher concentration (100µM) on *N. benthamiana* seedlings growth. *N. benthamiana* seedlings were exposed to either individual VOCs, hexadecane and 3-octanone, or control (DMSO) for 10 d and the dry weight of the seedlings was recorded. Error bars indicate the standard error of the mean (n = 3). Lowercase letters above the bars indicate a significant difference between treatments. Experiments were replicated at least three times producing similar results

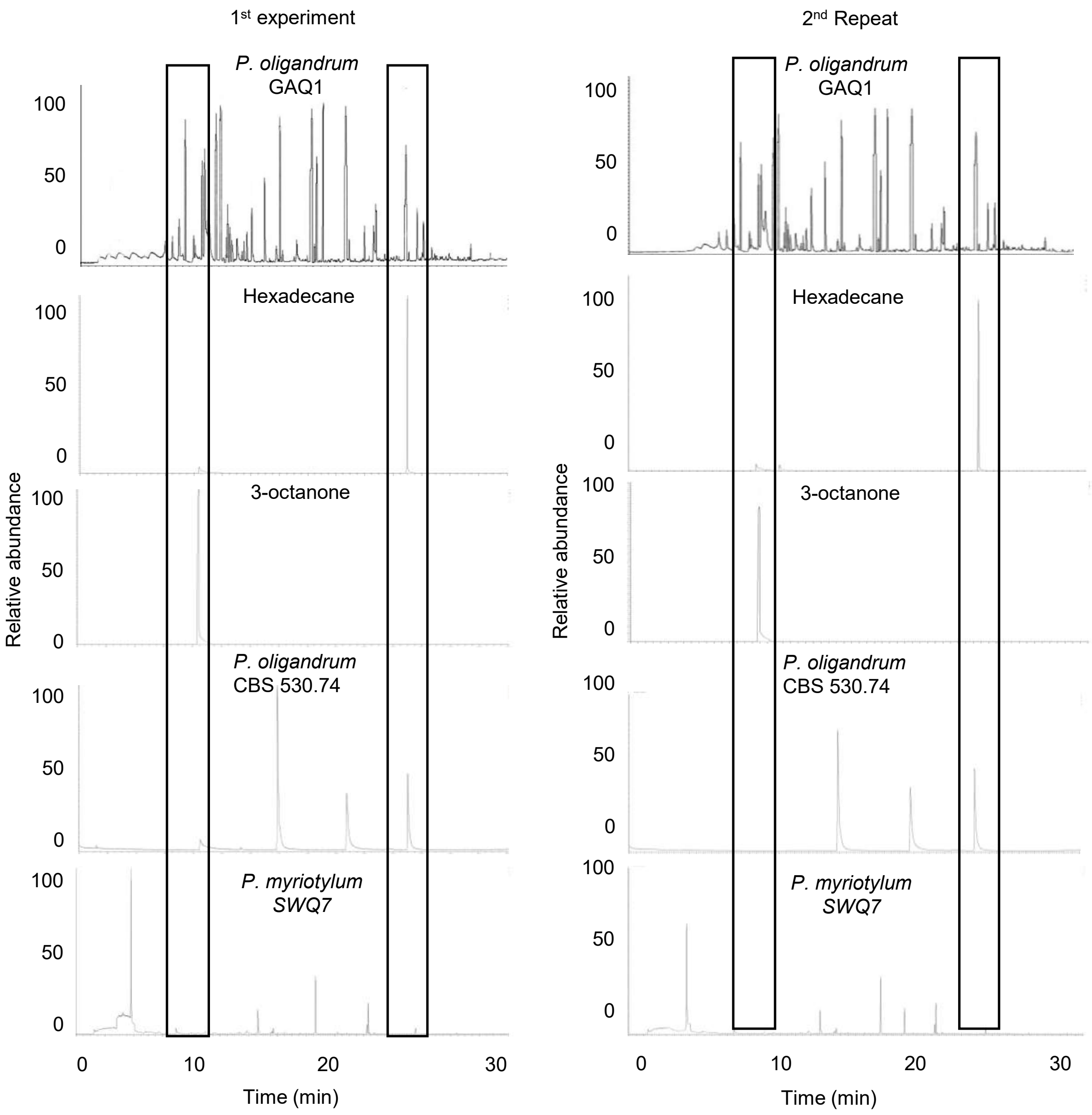

Figure S8. Chromatograph traces from GC-MS for the detection of the growth-promoting VOCs hexadecane and 3-octanone. The VOCs were produced by *P. oligandrum* GAQ1 on V8 media. The *P. oligandrum* strain CBS 530.74 also produced the VOC, hexadecane on the V8 medium whereas the two growth-promoting VOCs were not detected in *P. myriotylum* SWQ7 on the V8 medium. GC-MS traces are also shown for the standards of the two VOCs. The chromatographs of the second repeat of the experiment are also shown.

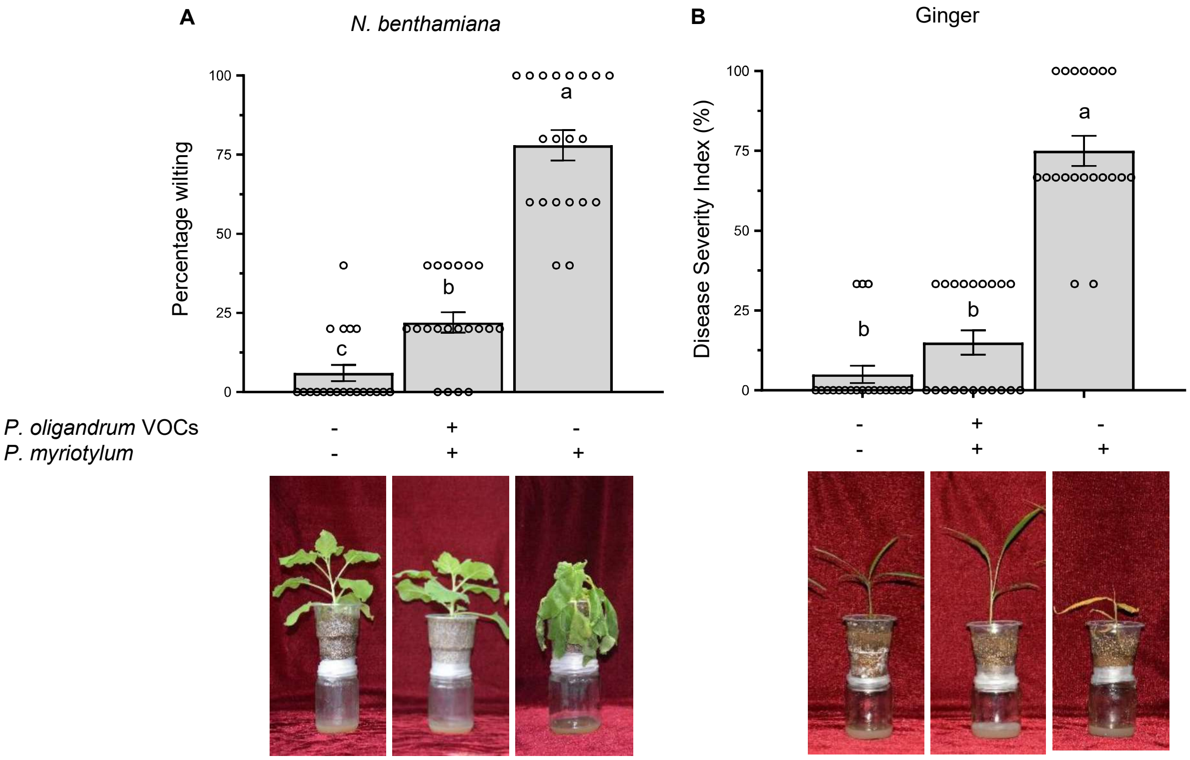

Figure S9. The results of the second repeat of the experiment also showed that *P. oligandrum* VOCs reduced the disease severity in *N. benthamiana* and ginger plants. **(A)** *P. myriotylum*-infected *N. benthamiana* plants were either exposed to control (V8) or Po-VOCs for 21 d, whereas the non-inoculated *N. benthamiana* plants without exposure to Po-VOCs were used as negative controls. The plants were photographed, and the symptoms were scored after 21 days of inoculation of *P. myriotylum* and exposure to Po-VOCs to calculate the percentage of wilting in *N. benthamiana*. **(B)** *P. myriotylum*-infected ginger plants were either exposed to Po-VOCs or control (V8) for 21 d, whereas the non-inoculated ginger plants without exposure to Po-VOCs were used as negative controls. The plants were photographed, and the symptoms were scored after 21 days of inoculation of *P. myriotylum* and exposure to Po-VOCs to calculate the disease severity index in ginger. Error bars are the standard error of the mean (n = 20). Lowercase letters on the bars show significant differences between different treatments.

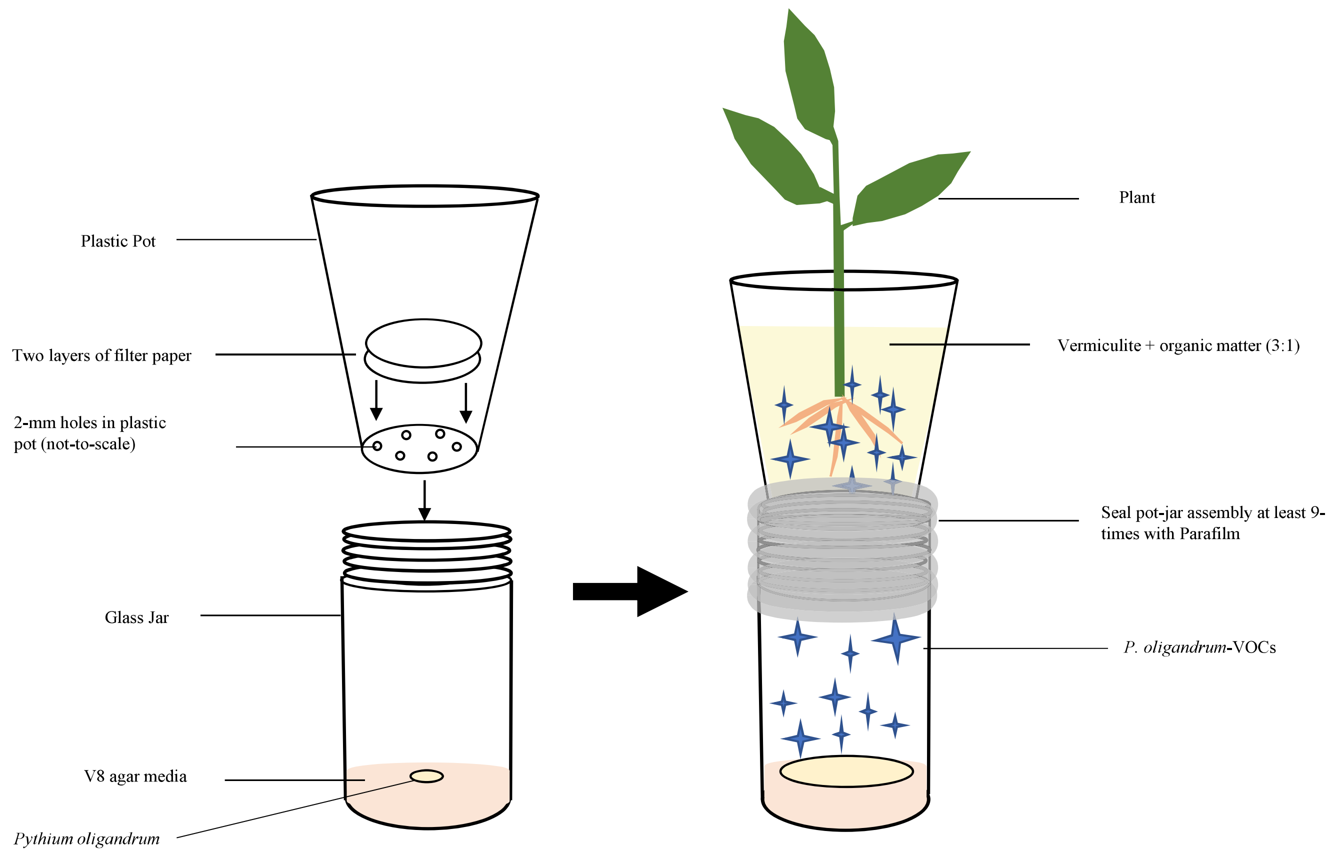

Figure S10. Pot-jar assembly used in the current study to see the effect of *P. oligandrum*-produced VOCs on plant growth, showing plastic pots with six small holes (2-mm) at the bottom to allow the plants to be exposed to the VOCs produced by *P. oligandrum.* Two layers of filter papers were placed inside at the bottom of the pot to avoid the leakage of contaminating liquid through the holes into the glass jars containing *P. oligandrum* grown on V8 agar medium. Plants were grown in the plastic pots containing vermiculite and organic matter (3:1) and fitted onto the glass jar to allow the plants to be exposed to the Po-VOCs. The glass jar assembly was sealed with Parafilm at least 9 times to avoid the escape of VOCs.
